# Female sex and cardiovascular disease risk in rural Uganda: a cross-sectional, population-based study

**DOI:** 10.1186/s12872-019-1072-9

**Published:** 2019-04-25

**Authors:** Itai M. Magodoro, Maggie Feng, Crystal M. North, Dagmar Vořechovská, John D. Kraemer, Bernard Kakuhikire, David Bangsberg, Alexander C. Tsai, Mark J. Siedner

**Affiliations:** 1 Harvard Medical School, 125 Shattuck St, Boston, MA 02115 USA; 2 Massachusetts General Hospital, Boston, MA USA; 3 Department of Health Systems Administration, Georgetown University, Washington, DC USA; 4 Mbarara University of Science & Technology, Mbarara, Uganda; 5 Oregon Health & Science University-Portland State University School of Public Health, Portland, Oregon, USA; 6 Africa Health Research Institute, Durban, KwaZulu-Natal South Africa

**Keywords:** Ideal cardiovascular health, Cardiovascular disease, Population health, Sex differences, Uganda, Sub-Saharan Africa

## Abstract

**Background:**

Sex-based differences in cardiovascular disease (CVD) burden are widely acknowledged, with male sex considered a risk factor in high-income settings. However, these relationships have not been examined in sub-Saharan Africa (SSA). We aimed to apply the American Heart Association (AHA) ideal cardiovascular health (CVH) tool modified by the addition of C-reactive protein (CRP) to examine potential sex-based differences in the prevalence of CVD risk in rural Uganda.

**Methods:**

In a cross-sectional study nested within a population-wide census, 857 community-living adults completed physical and laboratory-based assessments to calculate individual ideal CVH metrics including an eight category for CRP levels. We summarized sex-specific ideal CVH indices, fitting ordinal logistic regression models to identify correlates of improving CVH. As secondary outcomes, we assessed subscales of ideal CVH *behaviours* and *factors*. Models included inverse probability of sampling weights to determine population-level estimates.

**Results:**

The weighted-population mean age was 39.2 (1.2) years with 52.0 (3.7) % females. Women had ideal scores in smoking (80.4% vs. 68.0%; *p* < 0.001) and dietary intake (26.7% vs. 16.8%; *p* = 0.037) versus men, but the opposite in body mass index (47.3% vs. 84.4%; *p* < 0.001), glycated hemoglobin (87.4% vs. 95.2%; *p* = 0.001), total cholesterol (80.2% vs. 85.0%; *p* = 0.039) and CRP (30.8% vs. 49.7%; *p* = 0.009). Overall, significantly more men than women were classified as having optimal cardiovascular health (6–8 metrics attaining ideal level) (39.7% vs. 29.0%; *p* = 0.025). In adjusted models, female sex was correlated with lower CVH health factors sub-scales but higher ideal CVH behaviors.

**Conclusions:**

Contrary to findings in much of the world, female sex in rural SSA is associated with worse ideal CVH profiles, despite women having better indices for ideal CVH behaviors. Future work should assess the potential role of socio-behavioural sex-specific risk factors for ideal CVH in SSA, and better define the downstream consequences of these differences.

**Electronic supplementary material:**

The online version of this article (10.1186/s12872-019-1072-9) contains supplementary material, which is available to authorized users.

## Background

The exacting human cost imposed by cardiovascular diseases (CVDs) in both high and low-income settings has motivated innovative strategies to mitigate their impact. In 2010, the American Heart Association (AHA) [1] introduced the concept of “*ideal cardiovascular health* (ideal CVH)” in order to meet the urgent need for preventing cardiovascular morbidity and mortality. Ideal CVH is based on 7 metrics: smoking status, dietary intake, physical activity, body mass index (BMI), blood pressure (BP), total cholesterol (TC), and fasting blood glucose. When present at ideal levels, increasing frequency of these metrics is mirrored in proportionate decreases in risk of major adverse cardiovascular events (MACE) [2, 3].

Sex is a well-described independent risk factor for CVD [4, 5] with male sex considered a risk marker for incident atherosclerotic CVD in high-income settings [6]. This increased risk arises from both biological and sociocultural differences between men and women [7]. Sex as a biological variable underlies physiological variation in vascular function, coagulation, fibrinolysis and energy metabolism, among others [8, 9]. Gender also contributes to CVD risk through differences in social roles, environmental exposures, health seeking behaviors, and access to resources including medical care [4, 10, 11].

Whether and how these relationships between sex and gender and CVDs apply in sub-Saharan Africa (SSA) is not well known, despite the rapidly increasing burden of CVD in the region [12, 13]. Limited available evidence, however, indicates that women in SSA have a higher age-standardized CVD mortality rate than their male counterparts, and that this is substantially higher than the corresponding rates for both men and women in high-income countries [14]. Thus, an understanding of the role of sex and gender is critically important to curtail the human costs of CVDs and improve CVH in SSA.

To help build the evidence base to support the formulation of data-driven health policies in SSA, we aimed to define the AHA’s ideal CVH construct in a community-based, general population in rural Uganda. We hypothesized that the population distribution of ideal CVH metrics and indices would demonstrate lower rates of ideal CVH profiles than described elsewhere, and that these would differ between men and women.

## Methods

### Study population and setting

We conducted a cross-sectional study to describe AHA ideal CVH metrics in southwestern Uganda. Participants were community-dwelling adults, residing in Nyakabare Parish, Mbarara District, who attended one of five voluntary health fairs in June 2015. The parish is characterized by a subsistence pastoral-agrarian economy in which both food and water insecurity are common [15]. It is also the location of an ongoing longitudinal study that conducted a census in 2014 and collected data on 98% of all adults (1814/1851) residing there. Participation in the health fair study was limited to participants in the parent census study. Recruitment was enriched through radio advertisements and announcements at social and religious gatherings. The institutional review boards of Mbarara University of Science and Technology, Uganda, and Partners Healthcare, Boston, approved the protocol, and all study participants gave written informed consent. Consistent with national guidelines, we also obtained clearance for the study from the Ugandan National Council of Science and Technology and from the Research Secretariat in the Office of the President.

### Data collection

Surveys were administered to elicit age; sex; educational attainment; medical history; and active medication use for hypertension, dyslipidemia, heart failure, asthma or diabetes mellitus. We assessed socioeconomic status using household-owned assets and housing characteristics aggregated into an asset wealth index and divided in tertiles [16]. Questionnaires based on the International Physical Activity Questionnaire (IPAQ) [17] and the WHO STEPS instrument [18] were administered to collect data on physical activity, tobacco use, and fruit and vegetable intake. Physical activity was measured as metabolic equivalent of task (MET) in minutes per week [17].

We also measured height, weight, and BP. BP was measured in a seated position using automated sphygmomanometers (Omron HEM 705 LP, Omron Healthcare, Inc., Bannockburn, IL). Venous blood was collected to assess serum lipids, C-reactive protein (CRP), and performed at Epicentre Research Base in Mbarara, Uganda using a Cobas c111 (Roche, Basel, Switzerland) serum chemistry analyser and point-of-care glycated haemoglobin (HbA1c) (Siemens DCA Vantage, Munich, Germany).

### AHA cardiovascular health metrics

We categorized each of the seven CVH metrics defined by the AHA as poor, intermediate, or ideal based on published guidelines [1]. For our primary analyses, we added CRP as an additional metric, due to its strong correlation with CVD risk in multiple populations [19], allowing for a total of eight categories in our ideal CVH metrics score (Table 1). We also assigned two AHA sub-categories of ideal CVH, based on AHA recommendations, as: (1) five ideal CVH *factors*, which included BP, TC, HbA1c, CRP and BMI; and (2) three ideal CVH *behaviors,* which included diet, physical activity, and smoking.

**Table 1:** Definitions of American Heart Association Individual Ideal Cardiovascular Health Metrics

Metric	Definition	
	AHA Criteria	Criteria in present study
Total cholesterol (TC)		No adjustment
Ideal	TC < 200 mg/dL without use of any cholesterol-lowering medication	
Intermediate	TC 200–239 mg/dL or treated to < 200 mg/dL	
Poor	TC ≥240 mg/dL	
Blood pressure		No adjustment
Ideal	BP < 120/< 80 mmHg without use of antihypertensive medications	
Intermediate	Systolic BP 120–139 or diastolic BP 80–89 mmHg or treated to BP < 120/< 80 mmHg	
Poor	BP ≥140/≥90 mmHg	
Fasting plasma glucose		Glycated hemoglobin
Ideal	< 100 mg/dL	HbA1c < 5.7% and without any hypoglycemic medication
Intermediate	100–125 mg/dL	HbA1c 5.7–6.4% or treated to HbA1c < 5.7%
Poor	≥126 mg/dl	HbA1c ≥6.5%
Body mass index (BMI)		No adjustment
Ideal	BMI < 25 kg/m^2^	
Intermediate	BMI 25–29.9 kg/m^2^	
Poor	BMI ≥30 kg/m^2^	
Diet		No adjustment
Ideal	4–5 components	≥20 servings/week
Intermediate	2–3 components	
Poor	0–1 components	< 20 servings/week
Physical activity		No adjustment
Ideal	≥150 min/week moderate intensity or ≥75 min/week vigorous intensity or ≥150 min/week moderate + vigorous	≥1500METmin/week
Intermediate	1–149 min/week moderate intensity or 1–74 min/week vigorous intensity or 1–149 min/week moderate + vigorous	600-1500METmin/week
Poor	None	<600METmin/week
Smoking		No adjustment
Ideal	Never or quit > 12 months	Never having smoked or quit > 12 months prior
Intermediate	Former ≤12 months	Quit within the preceding 1–12 months
Poor	Current	Current smoking status or quit within the preceding 1 month.
C-reactive protein (CRP)	None	
Ideal		CRP < 1.0 mg/dL
Intermediate		CRP 1-3 mg/dL
Poor		CRP ≥3 mg/dL

### Ideal CVH health factors and health behaviors

We characterized the CVH metrics as poor, intermediate, or ideal as indicated in Table 1. In place of plasma fasting glucose in the standard AHA score, we measured HbA1c, and selected the three categories based on international consensus [20] HbA1c thresholds of < 5.7%, 5.7–6.4% and ≥ 6.5%, respectively, as cut-offs for normal, pre-diabetic, and diabetic states. CRP was categorized as ideal (< 1.0 mg/dL), intermediate (1-3 mg/dL), and poor (≥3 mg/dL) corresponding to standard definitions of CRP and CVD risk [21]. Complete data were available for only one of the five AHA components used to score the diet metric. We therefore used fruit and vegetable intake to define ideal (≥20 servings/week) and non-ideal (< 20 servings/week) diet as previously described [22]. Physical activity was converted to metabolic equivalents of task (METs) in minutes, and categorized as ≥1500 MET min/week for ideal, 600–1500 MET min/week for intermediate, and < 600 MET min/week for poor physical activity [17].

#### Data analysis

We used inverse probability of health fair attendance sampling weights to estimate population-representative descriptive statistics, cardiovascular health metrics, and regression models. To do so, we first estimated the probability of attending the health fair, conditional on participants` characteristics from the community census. This value was calculated by fitting logistic regression models with the entire census dataset, with health fair attendance as the outcome of interest and adjusted for 16 variables predicted to correlate with health fair attendance (See Additional file 1: Methods). This regression assigned a conditional probability weight of health fair attendance for each individual attending the health fair. The inverse of the predicted conditional probabilities of health fair attendance were then applied as stabilized inverse probability of treatment weights (IPTW) using methods described previously by Hernan et al. [23]. We assessed the validity of this method by comparing population characteristics as estimated by our IPTW models with variables in the census that were not included in the IPTW model (Additional file 1: Table S3).

We next summarized the weighted population dataset, applying stabilized IPTW weights, to obtain population-level characteristics and proportions assigned to each cardiovascular health index. Continuous variables were described by means and standard errors (SEs), and comparisons between groups were made using *t-*tests or corresponding nonparametric tests after assessing distributional properties. Categorical variables were described by percentages and compared by chi-squared tests.

Our primary outcome of interest was AHA ideal CVH metric score, which was categorized as an integer value ranging from 0 to 8 where participants received one point per criterion met for ideal health. As secondary outcomes of interest, we assessed ideal CVH behaviors as a score of 0–3 and ideal CVH factors as a score of 0–5. Our primary exposure of interest was sex. Secondary exposures of interest were age, categorized as 18–39; 40–59; and ≥ 60 years, wealth, categorized as tertiles of the Filmer-Pritchett asset index, and educational attainment. “Education was categorized as none”, “some primary education”, “completed primary education” and “post-primary education”.

We examined the distribution of each of the CVH metrics according to CVH status (poor, intermediate and ideal) stratified by sex and age. The numbers of health factors and health behaviors at the ideal level were also examined and graphically depicted by age and sex strata. Finally, we then fit univariable and multivariable ordinal logistic regression models to estimate adjusted associations between sex and indices of ideal cardiovascular health, ideal health behaviors and ideal health factors; and estimated the mean predicted probabilities of ideal CVH behaviors and factors by age and sex using post-estimation margins [24]. All statistical analyses were performed using Stata software (version 14.0, StataCorp, College Station, TX) with a two-sided *P*-value < 0.05 considered statistically significant.

## Results

### Demographic characteristics

A total of 857 individuals attended a health fair out of 1814 (47%) cohort participants. Analyses were confined to 756 (42%) participants with complete data to calculate all 7 AHA CVH metrics plus CRP. Compared to non-attendees, health fair attendees were older (*p* < 0.001), more likely to be female (p < 0.001), with less formal educational attainment (p < 0.001). Notably, attendees were twice as likely as non-attendees to report very bad or bad health (1.4% vs. 0.7, and 26.5% vs. 13.1%, respectively, p < 0.001) (Additional file 1: Table S4). There were no significant differences in cardiovascular risk characteristics between health fair attendees who were included or excluded from the analysis due to missing data (Additional file 1: Table S5).

The weighted study population mean age was 39.2 years (1.2), (Table 2). The population was 52.0% female, and 4.1 and 3.3%, respectively, had a history of heart failure or stroke. Compared to men, women were significantly older (41.5 vs. 36.8 years; *p* = 0.016), were less likely to have formal education (none: 20.1% vs. 5.2%), were more likely to be poor (44.2% vs. 29.1%), and had a higher BMI (mean BMI 26.0 vs. 22.4 kg/m2; *p* < 0.001). Three times as many women as men self-reported heart failure history (6.3% vs. 1.8%: *p* = 0.012).

**Table 2:** Baseline characteristics of weighted population

	Weighted population estimate, *mean (SE) or proportion (%) (SE)*			
	Female	Male	*P* value^*a*^	Total
Sex (%)	52.0 (3.7)	48.0 (3.7)	0.656	–
Age, mean (years)	41.5 (1.1)	36.8 (1.7)	**0.016**	39.2 (1.0)
Age group (years) (%)				
16–39	54.1 (3.0)	66.0 (4.3)		59.9 (2.8)
40–64	30.6 (2.6)	23.1 (3.3)		26.9 (2.2)
≥ 65	15.3 (1.9)	10.9 (2.0)	**0.042**	13.2 (1.4)
BMI (kg/m^2^)	26.0 (0.3)	22.4 (0.2)	**< 0.001**	24.3 (0.2)
Formal educational attainment (%)				
None	20.1 (2.0)	5.2 (1.3)		12.8 (1.3)
Some primary education	30.4 (2.5)	20.8 (3.1)		25.7 (2.1)
Completed primary education	21.8 (2.3)	24.4 (4.2)		23.1 (2.3)
Post-primary education	27.7 (3.4)	49.5 (5.7)	**< 0.001**	38.4 (3.6)
Filmer-Pritchett asset index (%)				
Poor	44.2 (2.9)	29.1 (4.2)		36.7 (2.7)
Middle	18.2 (2.0)	25.6 (6.1)		21.8 (3.2)
Rich	37.6 (3.1)	45.3 (5.7)	**0.047**	41.5 (3.2)
History of heart failure (%)	6.3 (1.1)	1.8 (0.1)	**0.012**	4.1 (0.7)
History of stroke (%)	1.8 (0.5)	4.8 (2.3)	**0.078**	3.3 (1.2)
Current hypertension (%)^*b*^	12.3 (1.8)	8.8 (1.8)	0.204	10.6 (1.4)
Systolic BP (mmHg)	123.7 (1.4)	124.5 (1.4)	0.564	124.0 (1.0)
Diastolic BP (mmHg)	80.1 (0.8)	76.7 (1.3)	**0.024**	78.5 (0.8)
Current diabetes mellitus^*c*^	2.2 (0.7)	1.6 (0.7)	0.546	2.0 (0.5)
HbA1c (%)	5.3 (0.04)	5.1 (0.03)	**> 0.001**	5.2 (0.03)
Total cholesterol (mg/dL)	167.0 (2.4)	155.7 (5.2)	**0.049**	161.5 (2.8)
Current use of chronic medication (%)	6.2 (1.2)	2.9 (0.1)	**0.037**	4.6 (0.8)

^a^Boldface indicates statistical significance (*p* < 0.05)

^b^Self-reported hypertension and/or blood pressure ≥ 140/90 mmHg at screening

^c^Self-reported diabetes mellitus and/or HbA1c ≥6.5%

### Cardiovascular health indices

**Fig. 1:**
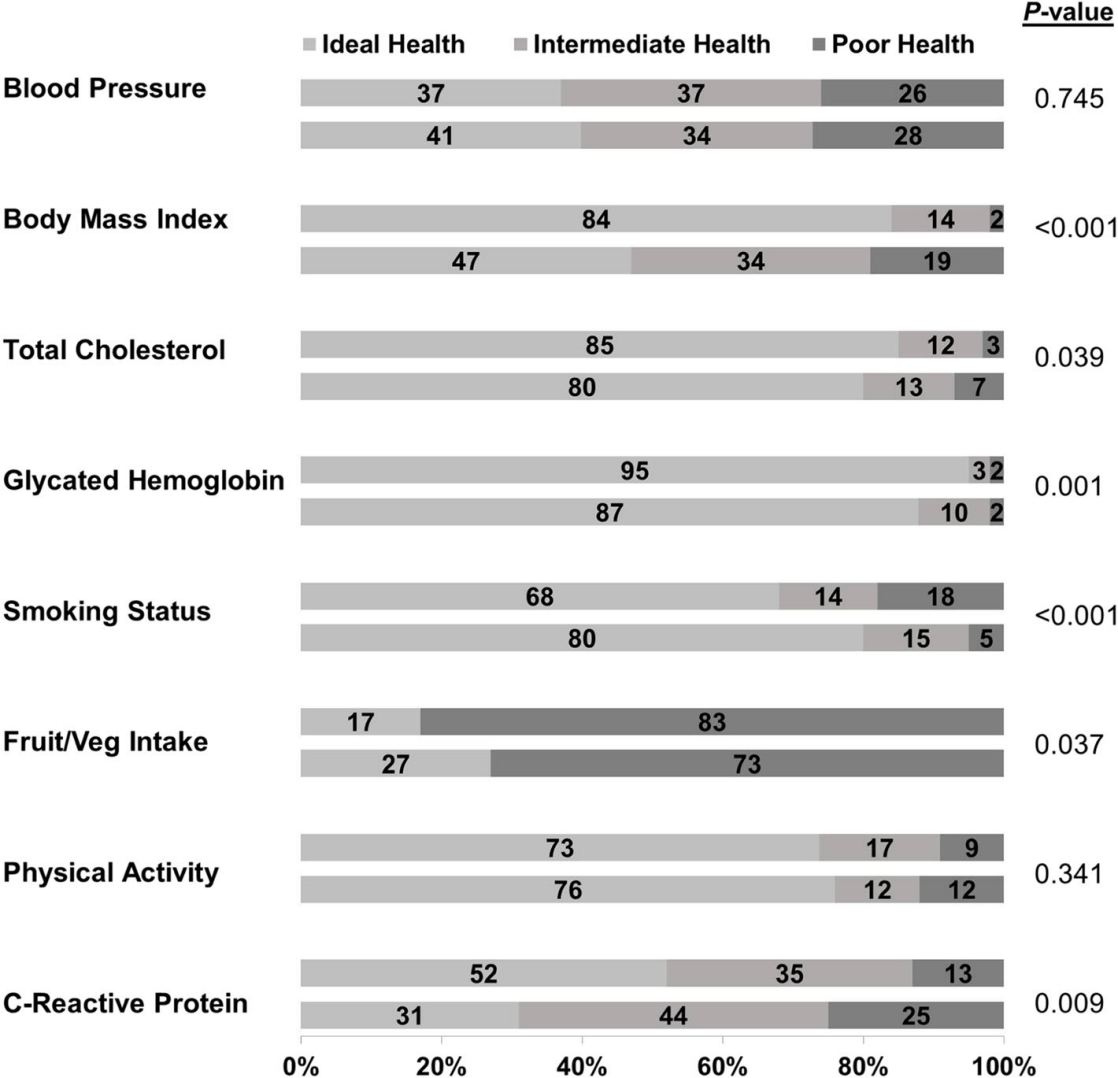
Prevalence of ideal, intermediate and poor cardiovascular health for each of the seven [7] metrics and C-reactive protein among males and females

**Fig. 2:**
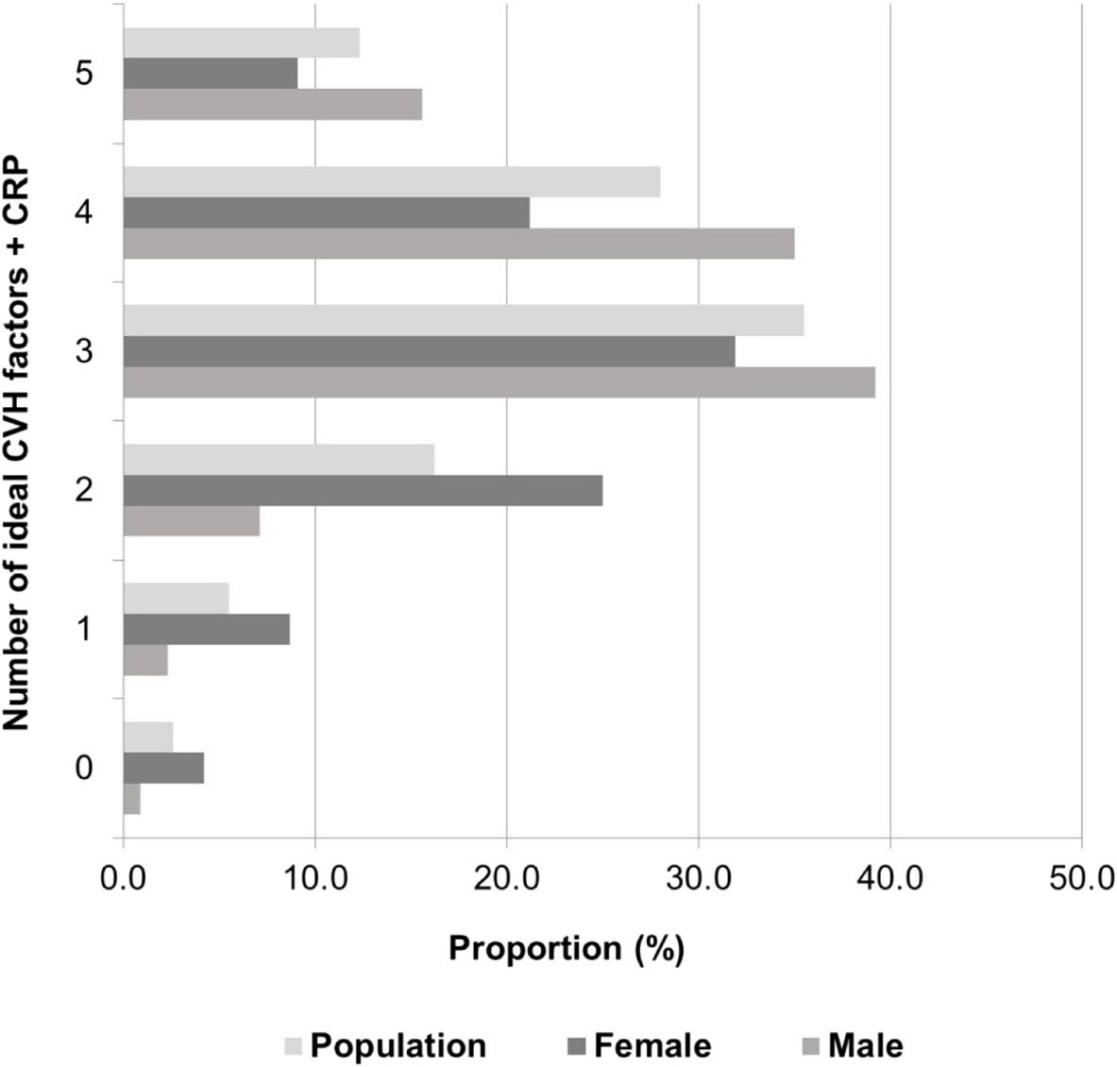
Weighted proportion of subjects with different numbers of ideal cardiovascular health factors in the overall population according to sex

**Fig. 3:**
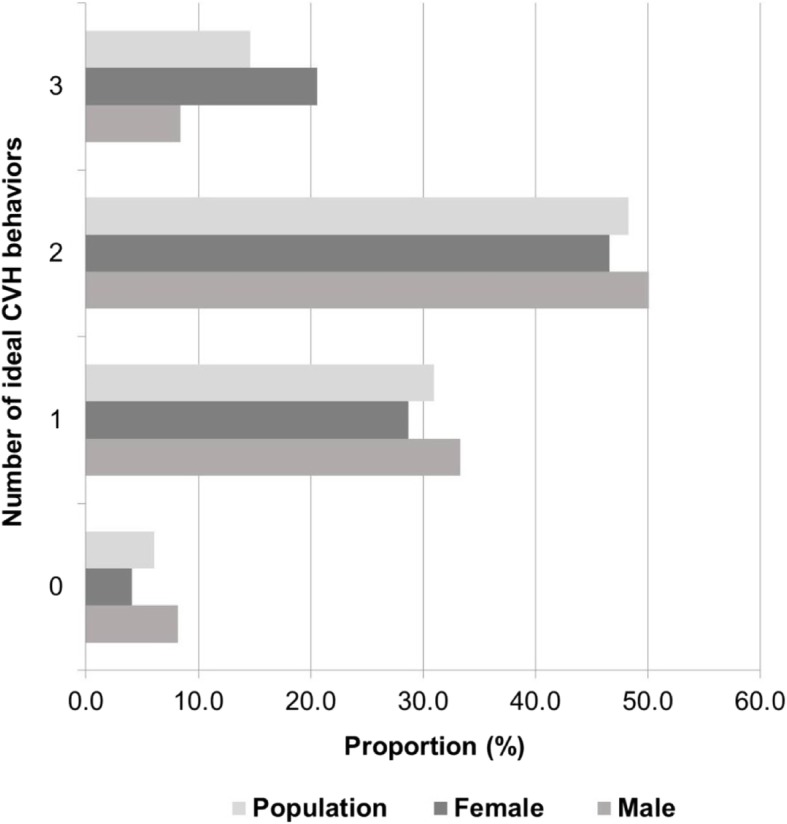
Weighted proportion of subjects with different numbers of ideal cardiovascular health behaviors in the overall population according to sex

The distribution of individual CVH metrics in the total population, and stratified by age and sex, are shown in Additional file 1: Table S1 and Fig. 1. Figures 2 and 3 depict the distribution of the number of ideal components of cardiovascular health, behaviors and factors by sex. Overall, only 3.2% of the weighted population had all 7 AHA metrics and CRP at ideal CVH levels. Though more women than men had all 8 metrics (CRP plus 7 AHA) at the ideal level (4.4% vs. 1.8%; *p* = 0.018), men had a higher number of metrics at ideal level out of 8 (5.1 vs. 4.7; *p* = 0.010) and higher proportion with at least 6 ideal CVH metrics (39.7% vs. 29.0%; *p* = 0.025) (Additional file 1: Table S2).

Men and women tended to have similar levels of ideal CVH categories for BP and physical activity (Fig. 1). However, men had a higher frequency of BMI, TC, HbA1c and CRP achieving ideal health status than the women. Overall, men had better CVH factor profiles (5 out of 5 metrics at ideal level) than women (16.3% vs. 9.4%; *p* < 0.001). However, women had the more favorable CVH behaviors profile (3 out of 3 metrics at ideal level) (8.8% vs. 20.4%; *p* = 0.027) (Figs. 2 and 3).

In univariable models, female sex was associated with worsening CVH status (OR 0.59; 95% CI: 0.40–0.87; *p* = 0.008) (Table 3). After adjusting for age, asset wealth and education, female sex remained correlated with worse CVH metrics, although this did not achieve statistical significance (adjusted OR 0.70; 95%CI: 0.47–1.03). Interestingly, women were more likely than men to be classified into higher categories of ideal CVH behaviors (adjusted OR 2.87; 95% CI: 1.72–4.78; *p* < 0.001), but less likely to be classified into higher categories of ideal CVH factors (adjusted OR 0.32; 95% CI: 0.21–0.49; p < 0.001) (Tables 4 and 5). In all models, increasing age was significantly associated with having fewer CVH metrics and factors. In both adjusted and unadjusted models, having some formal education, relative to no education, was positively correlated with ideal CVH behaviors. In contrast, relative household wealth was negatively associated with ideal CVH metrics and ideal health factors. Compared to the poorest quartile of asset ownership, increasing wealth predicted worsening CVH metrics and health factors. However, we found no apparent relationship between relative household wealth and health behaviors. The mean adjusted probabilities of having all 3 behaviors at the ideal CVH level, if age, wealth and educational attainment were equal to mean for all participants, were 18.0% (95% CI: 12.8–23.2%) for women and 7.1% (95% CI: 3.9–10.4) for men. The corresponding adjusted probabilities of having all 5 factors attaining ideal CVH level were 5.8% (95% CI: 3.1–8.6) for women and 16.0% (95% CI: 9.0–23.0) for men (Tables 6 and 7**)**.

**Table 3:** Association between ideal cardiovascular health metrics and demographic and socio-economic variables – ordered logistic regression

	Ideal CVH metrics					
	Univariate			Multivariable		
	OR	95% CI	*P* value^*a*^	OR	95% CI	*P* value^*a*^
Sex						
Male	1.00			1.0		
Female	0.59	0.40–0.87	**0.008**	0.70	0.47–1.03	0.072
Age group (years)						
16–39	1.00			1.00		
40–60	0.35	0.23–0.54	**< 0.001**	0.36	0.24–0.55	**< 0.001**
≥ 60	0.10	0.06–0.16	**< 0.001**	0.10	0.06–0.18	**< 0.001**
Filmer-Pritchett asset index						
Poor	1.00			1.00		
Middle	0.77	0.54–1.11	0.160	0.58	0.34–0.94	**0.028**
Rich	0.57	0.34–0.94	**0.027**	0.38	0.22–0.62	**< 0.001**
Highest educational attainment (%)						
None	1.0			1.0		
Some primary	2.17	1.40–3.37	**0.001**	1.39	0.84–2.30	0.195
Completed primary	2.95	1.81–4.80	**< 0.001**	1.51	0.83–2.73	0.177
Post-primary	3.40	1.97–5.88	**< 0.001**	1.99	1.08–3.66	**0.027**

^a^ Boldface indicates statistical significance (*p* < 0.05)

**Table 4:** Association between ideal cardiovascular health factors and demographic and socio-economic variables – ordered logistic regression

	Ideal CVH Factors					
	Univariate			Multivariable		
	OR	95% CI	*P* value^*a*^	OR	95% CI	*P* value^*a*^
Sex						
Male	1.00			1.0		
Female	0.34	0.23–0.52	**< 0.001**	0.32	0.21–0.49	**< 0.001**
Age group (years)						
16–39	1.00			1.00		
40–60	0.42	0.28–0.63	**< 0.001**	0.41	0.27–0.62	**< 0.001**
≥ 60	0.25	0.15–0.40	**< 0.001**	0.21	0.13–0.35	**< 0.001**
Filmer-Pritchett asset index						
Poor	1.00			1.00		
Middle	0.61	0.41–0.91	**0.014**	0.47	0.26–0.86	**0.015**
Rich	0.44	0.26–0.75	**0.003**	0.31	0.18–0.53	**< 0.001**
Highest educational attainment (%)						
None	1.0			1.0		
Some primary	1.46	0.96–2.22	**0.075**	1.01	0.64–1.61	0.958
Completed primary	2.08	1.25–3.46	**0.003**	1.15	0.67–1.97	0.614
Post-primary	1.84	1.08–3.12	**0.024**	1.08	0.56–2.08	0.826

^a^ Boldface indicates statistical significance (*p < 0.05)

**Table 5:** Association between ideal cardiovascular health behaviors and demographic and socio-economic variables – ordered logistic regression

Characteristic	Ideal CVH Behaviors					
	Univariate			Multivariable		
	OR	95% CI	*P* value^*a*^	OR	95% CI	*P* value^*a*^
Sex						
Male	1.00			1.0		
Female	1.76	1.14–2.72	**< 0.001**	2.87	1.72–4.78	**< 0.001**
Age group (years)						
16–39	1.00			1.00		
40–60	0.57	0.37–0.88	**0.011**	0.56	0.36–0.88	**0.011**
≥ 60	0.12	0.07–0.21	**< 0.001**	0.15	0.08–0.27	**< 0.001**
Filmer-Pritchett asset index						
Poor	1.00			1.00		
Middle	1.14	0.71–1.82	0.585	1.04	0.66–1.64	0.858
Rich	1.05	0.64–1.71	0.860	0.86	0.52–1.41	0.557
Highest educational attainment (%)						
None	1.0			1.0		
Some primary	2.60	1.50–4.48	**0.001**	1.95	1.11–3.43	**0.020**
Completed primary	2.92	1.57–5.42	**0.001**	1.95	0.97–3.92	0.059
Post-primary	3.98	2.14–7.39	**< 0.001**	3.38	1.67–6.84	**0.001**

^a^ Boldface indicates statistical significance (*p < 0.05)

**Table 6:** Adjusted probability of ideal cardiovascular health metrics by demographic and socio-economic characteristics

	Adjusted predicted probability, *% (95% CI)*				
	Number of ideal CVH factors				
	≤1	2	3	4	5
Mean overall probability	6.0 (4.3–7.5)	14.9 (12.1–17.8)	41.4 (34.1–48.7)	28.3 (22.0–34.5)	9.4 (5.6–13.3)
Sex					
Female	9.7 (6.7–12.7)	21.2 (16.4–26.0)	42.4 (35.9–49.0)	20.8 (14.7–26.9)	5.8 (3.1–8.6)
Male	3.4 (2.2–4.6)	9.3 (6.8–11.9)	34.6 (26.1–43.0)	36.7 (29.3–44.1)	16.0 (9.0–23.0)
Age group (years)					
16–39	3.8 (2.5–5.0)	10.3 (7.8–12.8)	37.3 (28.9–45.6)	35.3 (27.8–42.9)	14.5 (8.1–20.9)
40–60	8.6 (5.7–11.5)	19.5 (14.3–24.7)	42.5 (35.8–49.3)	22.7 (16.7–28.8)	6.6 (3.7–9.6)
≥ 60	15.7 (9.1–22.3)	28.0 (21.4–34.5)	39.0 (32.1–46.0)	13.9 (7.8–19.9)	3.5 (1.4–5.6)
Filmer-Pritchett asset index					
Poor	3.1 (1.9–4.3)	8.6 (5.9–11.3)	33.3 (25.9–40.6)	37.7 (30.4–45.0)	17.3 (10.6–23.9)
Middle	6.4 (2.8–9.9)	15.7 (9.8–21.5)	41.5 (32.7–50.2)	27.6 (16.2–38.9)	8.9 (3.6–14.3)
Rich	9.4 (5.9–12.8)	20.7 (14.8–26.6)	42.5 (35.9–49.1)	21.3 (14.5–28.2)	6.1 (2.5–9.6)
Highest educational attainment (%)					
None	6.3 (3.6–9.1)	15.6 (10.2–21.0)	41.4 (33.8–49.1)	27.6 (19.1–36.1)	9.0 (4.1–13.8)
Some primary	6.2 (4.0–8.3)	15.3 (11.4–19.2)	41.2 (34.4–48.1)	28.1 (21.8–34.4)	9.2 (5.6–12.8)
Completed primary	5.3 (3.2–7.5)	13.7 (9.1–18.4)	40.1 (33.32–47.0	30.3 (23.2–37.2)	10.5 (5.9–15.1)
Post-primary	5.7 (3.0–8.3)	14.4 (9.5–19.2)	40.6 (31.5–49.8)	29.4 (19.6–39.1)	10.0 (3.8–16.1)

**Table 7:** Adjusted probability of ideal cardiovascular health behaviors by demographic and socio-economic characteristics

	Adjusted predicted probability, *% (95% CI)*			
	Number of ideal cardiovascular health behaviors			
	0	1	2	3
Mean overall probability	4.3 (2.7–5.8)	31.6 (25.3–37.9)	52.7 (45.3–60.1)	11.4 (8.0–14.9)
Sex				
Female	2.6 (1.4–3.9)	42.3 (34.2–50.4)	56.5 (48.7–64.3)	18.0 (12.8–23.2)
Male	7.2 (4.4–10.0)	22.8 (15.7–30.0)	43.4 (35.3–51.4)	7.1 (3.9–10.4)
Age group (years)				
16–39	2.9 (1.6–4.2)	24.6 (17.3–31.9)	56.0 (48.1–63.8)	16.5 (11.3–21.7)
40–60	5.21 (2.9–7.2)	35.2 (28.2–42.3)	49.7 (42.2–57.1)	10.0 (6.0–14.0)
≥ 60	17.1 (9.6–24.5)	55.1 (47.7–62.5)	25.0 (15.4–34.6)	2.8 (1.0–4.6)
Filmer-Pritchett asset index				
Poor	4.1 (2.2–6.0)	31.1 (23.5–38.8)	52.6 (44.8–60.4)	12.1 (7.3–16.9)
Middle	4.0 (2.2–5.8)	30.4 (23.5–37.2)	53.1 (45.1–61.1)	12.6 (8.7–16.4)
Rich	4.8 (2.7–6.8)	34.0 (24.4–43.6)	50.6 (41.5–59.7)	10.6 (6.1–15.0)
Highest educational attainment (%)				
None	9.2 (4.3–14.1)	46.9 (36.7–57.1)	38.3 (27.2–49.4)	5.5 (2.1–8.9)
Some primary	4.9 (2.9–7.0)	34.6 (27.8–41.4)	50.1 (42.7–57.5)	10.3 (6.3–14.3)
Completed primary	4.9 (2.5–7.4)	34.6 (25.4–43.9)	50.1 (41.4–58.9)	10.3 (5.4–15.2)
Post-primary	2.9 (1.4–4.4)	24.6 (15.3–33.8)	56.0 (47.7–64.2)	16.6 (10.3–22.8)

## Discussion

In this cross-sectional, population-based study in rural Uganda, we found significant sex-based differences in CVH metrics. Based on a modified definition of AHA ideal CVH with 8 metrics, significantly more men than women were classified as having optimum CVH (6–8 metrics attaining ideal level) (39.7% vs. 29.0%; *p* = 0.025), while 3 times as many women as men had poor CVH (0–2 metrics attaining ideal level) (7.8% vs. 2.9%; p = 0.025). Additionally, women had significantly worse CVH factor profiles despite having significantly increased CVH behavior profiles. These relationships remained consistent after adjustment for age, household wealth and educational attainment.

The sex differences in ideal CVH were unexpected, and contrary to what has been reported in high-income areas. For example, in the Heart SCORE study [25], reporting on ideal CVH among community-living adults (mean age 59 years) of mixed ethnicity/race in the USA, women had better CVH factor profiles than men but similar CVH behavior profiles. Similarly, in an urban center in Northern China [26], the proportion of women with 6–7 ideal CVH metrics was 11-fold higher than that of their male counterparts (3.42% vs. 0.31%; *p* < 0.001). A similar trend has also been reported in rural China [27]. Nonetheless, an increased risk for cardiovascular deaths among women versus men in SSA consistent with our results was also demonstrated in a study by Mensah *et. al.,* [14]. If this data is corroborated with prospective data including outcomes, it would argue for a reconsideration of male sex as a primary risk factor for MACE in the region.

The sex-based differences in ideal CVH we noted were driven partially by elevated levels of CRP, which were present in most women. Systemic inflammation is an important pathophysiological mechanism underlying CVD, and increased CRP levels are independently predictive of MACE in western populations [28]. Systemic inflammation has complex associations with the AHA metrics. Smoking, diet and physical activity, for example, are causally related to inflammation [29–31]. However, the fact that women in our study and others in Uganda had better health behaviors and worse inflammatory profiles than the men, suggests the presence of an alternative mechanism for increased inflammation in this population.

One possible cause for these differences in systemic inflammation may be chronic exposure to biomass fuel combustion. Wood and other solid biomass fuels are the main energy sources for cooking in many households in SSA. Cooking in Ugandan villages is generally done by girls and women, and often in poorly ventilated kitchens [32]. Thus exposure to polluted indoor air begins in early life for many women. Biomass gases cause systemic inflammation and oxidative stress, which may be the mechanistic pathways involved in CVD development [33–35]. One study in rural India recently reported greater prevalence of hypertension among relatively young, never-smoking housewives who cooked exclusively with biomass compared to age-, sex- and community-matched peers using alternative energy sources [36]. This hypothesis remains to be tested in SSA, where 81% of households use biomass fuels as their primary energy source [37].

In addition to worse ideal CVH profiles among women, our findings also indicate a relatively high prevalence of ideal CVH in rural Uganda compared to other populations. We estimated that approximately 1 in 5 individuals (22.3%) of our study population had at least 6 AHA standard metrics at ideal level, which is at least twice as high as the prevalence in other comparably young populations. For example, Ogunmoroti *et. al.* (2015) [38], reported a prevalence of 12% for ideal CVH (≥6 ideal metrics) in a US-based population with mean age 43 years. The prevalence was much lower at 0.3% in an Iranian population with a mean age 41 years [39].

We also found an unexpected relationship between household wealth and CVD risk profiles. The protective effect of increasing economic wellbeing on CVD risk is well established in many studies [40, 41]. In contrast, we found that increasing household wealth was correlated with worsening ideal CVH health factors. This finding warrants further exploration [42, 43].

### Strengths and limitations

There are a number of limitations to our study. The absence of longitudinal data on ideal CVH metrics in our study population prevents us from estimating temporal trends in CVD risk, and confirming whether CVD risk factors predict CVH morbidity or mortality. The lack of population-based cardiovascular outcomes data in Uganda largely precludes such estimation of the clinical and population health implications of our findings. As a cross-sectional study, we are further limited to correlational inferences between ideal CVH metrics, sex/gender and social determinants. Finally, our study used definitions of dietary intake and glucose metabolism that differed than those recommended by the AHA. This should be considered when making direct comparisons with other studies of ideal CVH metrics.

Our study also had important strengths. As a relatively large population-based study, with participants drawn from a complete census, we were able to make population level estimates and generalize our results to similar areas of rural Uganda. Differential participation in the health fairs was accounted for with the use of IPTW-adjusted models to derive population-level estimates. We were also able to include both unique biomedical factors, such as CRP, and social determinants of health, such as wealth, education, and sex to contextualize our findings beyond basic CVD risk estimation.

## Conclusions

In this analysis, we present one of the first reports of AHA ideal CVH metrics from SSA. We demonstrated that women had worse CVH factors, despite having better CVH behaviors than men, and that age, wealth, and education all contribute to CVD risk in this setting. Our findings highlight additional areas to be prioritized for further study, including the downstream repercussions of these relationships between sex and ideal CVH profiles, and the potential role of behavioral exposures, such as biomass fuel, as sex-specific CVD risk factors.

## Additional file

Additional file 1: **Table 1:** Distribution of individual cardiovascular health metrics for the weighted population. **Table 2:** Prevalence of ideal cardiovascular health metrics according to age and sex. **Table 3:** Comparison of population estimates based on weightings from inverse probability of health fair attendance models versus true population statistics. **Table 4:** Characteristics of Health Fair Attendees versus Non-attendees. **Table 5:** Comparison of characteristics of participants with and without complete AHA metrics data. **Methods:** Description of variables used in the inverse probability of treatment weights (IPTW). (DOCX 33 kb)

## Abbreviations

AHA: American Heart Association
BMI: Body mass index
CRP: C - reactive protein
CVD: Cardiovascular disease
CVH: Cardiovascular health
IPAQ: International physical activity questionnaire
IPTW: Inverse probability of treatment weights
MACE: Major adverse cardiovascular events
MET: Metabolic equivalent of task
SSA: Sub-Saharan Africa
STEPS: STEPwise approach to surveillance
WHO: World Health Organization

## References

[1] Lloyd-Jones DM, Hong Y, Labarthe D, Mozaffarian D, Appel LJ, Van Horn L (2010). Defining and setting National Goals for cardiovascular Health promotion and disease reduction: the American Heart Association’s strategic impact goal through 2020 and beyond. Circulation..

[2] Ommerborn MJ, Blackshear CT, Hickson DMA, Griswold ME, Kwatra J, Djoussé L (2016). Ideal cardiovascular Health and incident cardiovascular events: the Jackson heart study. Am J Prev Med [Internet]..

[3] Foraker RE, Abdel-Rasoul M, Kuller LH, Jackson RD, Van Horn L, Seguin RA, et al. Cardiovascular Health and incident cardiovascular disease and Cancer: the Women’s Health initiative. Am J Prev Med [Internet] 2016;50(2):236–240. Available from: 10.1016/j.amepre.2015.07.039 10.1016/j.amepre.2015.07.039 PMC4718741 26456876

[4] O’Neil A, Scovelle AJ, Milner AJ, Kavanagh A. Gender/sex as a social determinant of cardiovascular risk. Circulation. 2018;137(8):854–64. https://www.ahajournals.org/doi/10.1161/CIRCULATIONAHA.117.028595. 10.1161/CIRCULATIONAHA.117.028595 29459471

[5] Regitz-Zagrosek V, Oertelt-Prigione S, Prescott E, Franconi F, Gerdts E, Foryst-Ludwig A (2016). Gender in cardiovascular diseases: impact on clinical manifestations, management, and outcomes. Eur Heart J.

[6] Lloyd-Jones DM, Wilson PWF, Larson MG, Beiser A, Leip EP, D’Agostino RB (2004). Framingham risk score and prediction of lifetime risk for coronary heart disease. Am J Cardiol.

[7] Vlassoff C (2007). Gender differences in determinants and consequences of Health and illness. J Health Popul Nutr.

[8] Heidari S, Babor TF, De Castro P, Tort S, Curno M. Sex and gender equity in research: rationale for the SAGER guidelines and recommended use. Res Integr Peer Rev [Internet]. 2016;1(1):2. Available from: https://researchintegrityjournal.biomedcentral.com/articles/10.1186/s41073-016-0007-6. 10.1186/s41073-016-0007-6 PMC5793986 29451543

[9] Ahonen TM, Kautiainen HJ, Keinänen-Kiukaanniemi SM, Kumpusalo EA, Vanhala MJ (2008). Gender difference among smoking, adiponectin, and high-sensitivity C-reactive protein. Am J Prev Med [Internet]..

[10] Walters V. The social context of Women’s Health. BMC Womens Health [Internet]. 2004;4(1):S2. https://bmcwomenshealth.biomedcentral.com/articles/10.1186/1472-6874-4-S1-S2. 10.1186/1472-6874-4-S1-S2 PMC2096697 15345065

[11] Perl L, Peiffer V, Fuhrer AE, D’Ascenzo F, Pietzsch JB (2018). Sex differences in discharge destination following acute myocardial infarction. Coron Artery Dis.

[12] Magodoro IM, Esterhuizen TM, Chivese T. A cross-sectional, facility based study of comorbid non-communicable diseases among adults living with HIV infection in Zimbabwe. BMC Res Notes. 2016;9(1). 10.1186/s13104-016-2187-z PMC4969634 27484005

[13] Keates AK, Mocumbi AO, Ntsekhe M, Sliwa K, Stewart S. Cardiovascular disease in Africa: epidemiological profile and challenges. Nat Rev Cardiol [Internet]. 2017;14(5):273–293. Available from: 10.1038/nrcardio.2017.19 10.1038/nrcardio.2017.19 28230175

[14] Mensah GA, Sampson UK, Roth GA, Forouzanfar MH, Naghavi M, Murray CJ, Moran AE, Feigin VL. Mortality from cardiovascular diseases in sub-Saharan Africa, 1990–2013: a systematic analysis of data from the Global Burden of Disease Study 2013. Cardiovascular journal of Africa. 2015;26(2 H3Africa Suppl):S6. http://www.cvja.co.za/onlinejournal/vol26/vol26_issue2_supplement/files/assets/basic-html/page-8.html. 10.5830/CVJA-2015-036 PMC4557490 25962950

[15] Tsai AC, Bangsberg DR, Emenyonu N, Senkungu JK, Martin JN, Weiser SD (2011). The social context of food insecurity among persons living with HIV/AIDS in rural Uganda. Soc Sci Med [Internet].

[16] Filmer D, Pritchett LH. Estimating wealth effects without expenditure data—or tears: an application to educational enrollments in states of India. Demography. 2001;38(1):115–32. https://link.springer.com/article/10.1353%2Fdem.2001.0003. 10.1353/dem.2001.0003 11227840

[17] Craig CLC, Marshall AL, Sjostrom M, Sj??str??m M, Bauman AE, Booth ML, et al. International physical activity questionnaire: 12 country reliability and validity. Med Sci Sport Exerc 2003;35(August):1–7. 10.1249/01.MSS.0000078924.61453.FB 12900694

[18] Riley L, Guthold R, Cowan M, Savin S, Bhatti L, Armstrong T (2016). The World Health Organization STEPwise approach to noncommunicable disease risk-factor surveillance: methods, challenges, and opportunities. Am J Public Health.

[19] Rohde LE, Hennekens CH, Ridker PM. Survey of C-reactive protein and cardiovascular risk factors in apparently healthy men. Am J Cardiol. 1999;84(9):1018–22. https://www.ajconline.org/article/S0002-9149(99)00491-9/fulltext. 10.1016/s0002-9149(99)00491-9 10569656

[20] American Diabetes Association. Classification and diagnosis of diabetes: standards of medical care in diabetes—2018. Diabetes care. 2018;41(Supplement 1):S13–S27. http://care.diabetesjournals.org/content/41/Supplement_1/S13. 10.2337/dc18-S002 29222373

[21] Salazar J, Martínez MS, Chávez M, Toledo A, Añez R, Torres Y, Apruzzese V, Silva C, Rojas J, Bermúdez V. C-reactive protein: clinical and epidemiological perspectives. Cardiology research and practice; 2014. https://www.hindawi.com/journals/crp/2014/605810/. 10.1155/2014/605810 PMC3932642 24653858

[22] Feinstein MJ, Kim JH, Bibangambah P, Sentongo R, Martin JN, Tsai AC, Bangsberg DR, Hemphill L, Triant VA, Boum Y, Hunt PW. Ideal cardiovascular Health and carotid atherosclerosis in a mixed cohort of HIV-infected and uninfected Ugandans. AIDS Res Hum Retrovir [Internet]. 2017;33(1):49–56. https://www.liebertpub.com/doi/10.1089/aid.2016.0104. 10.1089/aid.2016.0104 PMC5240009 27476547

[23] Hernán MA, Robins JM (2006). Estimating causal effects from epidemiological data. J Epidemiol Community Health.

[24] Williams R (2012). Using the margins command to estimate and interpret adjusted predictions and marginal effects. Stata J.

[25] Bambs C, Kip KE, Dinga A, Mulukutla SR, Aiyer AN, Reis SE (2011). Low prevalence of ideal cardiovascular Health in a community-based population: the heart strategies concentrating on risk evaluation (heart SCORE) study. Circulation..

[26] Wu S, Huang Z, Yang X, Zhou Y, Wang A, Chen L (2012). Prevalence of ideal cardiovascular Health and its relationship with the 4-year cardiovascular events in a northern Chinese Industrial City. Circ Cardiovasc Qual Outcomes.

[27] Chang Y, Guo X, Chen Y, Guo L, Li Z, Yu S (2016). Prevalence and metrics distribution of ideal cardiovascular Health: a population-based, cross-sectional study in rural China. Hear Lung Circ [Internet].

[28] Haffner SM. The metabolic syndrome: inflammation, diabetes mellitus, and cardiovascular disease. Am J Cardiol. 2006;97(2 SUPPL. 1). 10.1016/j.amjcard.2005.11.010 16442931

[29] Abramson JL, Vaccarino V (2002). Relationship between physical activity and inflammation among apparently healthy middle-aged and older US adults. Arch Intern Med.

[30] Kasapis C, Thompson PD (2005). The effects of physical activity on serum C-reactive protein and inflammatory markers: a systematic review. J Am Coll Cardiol [Internet].

[31] Galland L (2010). Diet and inflammation. Nutr Clin Pract.

[32] Muyanja D, Allen JG, Vallarino J, Valeri L, Kakuhikire B, Bangsberg DR (2017). Kerosene lighting contributes to household air pollution in rural Uganda. Indoor Air.

[33] Olopade CO, Frank E, Bartlett E, Alexander D, Dutta A, Ibigbami T, et al. Effect of a clean stove intervention on inflammatory biomarkers in pregnant women in Ibadan, Nigeria: a randomized controlled study. Environ Int [Internet]. 2017;98:181–190. Available from: 10.1016/j.envint.2016.11.004 10.1016/j.envint.2016.11.004 27839852

[34] Neupane M, Basnyat B, Fischer R, Froeschl G, Wolbers M, Rehfuess EA (2015). Sustained use of biogas fuel and blood pressure among women in rural Nepal. Environ Res [Internet].

[35] Dutta A, Ray MR, Banerjee A (2012). Systemic inflammatory changes and increased oxidative stress in rural Indian women cooking with biomass fuels. Toxicol Appl Pharmacol [Internet].

[36] Dutta A, Mukherjee B, Das D, Banerjee A, Ray MR. Hypertension with elevated levels of oxidized low‐density lipoprotein and anticardiolipin antibody in the circulation of premenopausal Indian women chronically exposed to biomass smoke during cooking. Indoor air. 2011;21(2):165–76. https://onlinelibrary.wiley.com/doi/abs/10.1111/j.1600-0668.2010.00694.x. 10.1111/j.1600-0668.2010.00694.x 21118307

[37] Venkataraman C, Sagar AD, Habib G, Lam N, Smith KR. The Indian national initiative for advanced biomass cookstoves: the benefits of clean combustion. Energy Sustain Dev. 2010;14(2):63–72. https://www.sciencedirect.com/science/article/pii/S0973082610000219?via%3Dihub.

[38] Ogunmoroti O, Utuama O, Spatz ES, Rouseff M, Parris D, Das S (2016). Trends in ideal cardiovascular Health metrics among employees of a large healthcare organization (from the Baptist Health South Florida employee study). Am J Cardiol.

[39] Moghaddam MM, Mohebi R, Hosseini F, Lotfaliany M, Azizi F, Saadat N (2014). Distribution of ideal cardiovascular Health in a community-based cohort of Middle East population. Ann Saudi Med.

[40] National Center for Health Statistics US, 2010. Health, United States, 2009: With special feature on medical technology. https://www.cdc.gov/nchs/data/hus/hus09.pdf. 20698070

[41] Vissandjee B, Desmeules M, Cao Z, Abdool S (2004). Integrating socio-economic determinants of Canadian Women’s Health. BMC Womens Health.

[42] Riha J, Karabarinde A, Ssenyomo G, Allender S, Asiki G, Kamali A, et al. Urbanicity and lifestyle risk factors for Cardiometabolic diseases in rural Uganda: a cross-sectional study. PLoS Med. 2014;11(7). 10.1371/journal.pmed.1001683 PMC4114555 25072243

[43] Liburd LC, Jack L, Williams S, Tucker P (2005). Intervening on the social determinants of cardiovascular disease and diabetes. Am J Prev Med.

