# Supplementary material for: Female sex and cardiovascular disease risk in rural Uganda: a cross-sectional, population-based study
Source: BMC Cardiovasc Disord. 2019 Apr 25;19:96. doi: 10.1186/s12872-019-1072-9 (PMC6485175; doi:10.1186/s12872-019-1072-9)
Supplement: Supplementary file 1 — Table 1: Distribution of individual cardiovascular health metrics for the weighted population. Table 2: Prevalence of ideal cardiovascular health metrics according to age and sex. Table 3: Comparison of population estimates based on weightings from inverse probability of health fair attendance models versus true population statistics. Table 4: Characteristics of Health Fair Attendees versus Non-attendees. Table 5: Comparison of characteristics of participants with and without complete AHA metrics data. Methods: Description of variables used in the inverse probability of treatment weights (IPTW). (DOCX 33 kb) [file 12872_2019_1072_MOESM1_ESM.docx]

Additional Table 1: Distribution of individual cardiovascular health metrics for the weighted population

|  | Weighted population estimates, *proportions (SE)* | | | | | | |
| --- | --- | --- | --- | --- | --- | --- | --- |
|  | Sex | | | Age group (years) | | | |
|  | Female | Male | P value*^a^* | 16-39 | 40-64 | ≥65 | P value*^a^* |
| *BMI (kg/m2)* |  |  |  |  |  |  |  |
| Ideal (<25) | 47.3 (3.0) | 86.4 (3.2) |  | 69.9 (3.9) | 59.3 (3.8) | 65.5 (4.8) |  |
| Intermediate (25-29.9) | 33.4 (2.9) | 11.6 (3.2) |  | 21.7 (3.4) | 25.8 (3.4) | 21.3 (4.3) |  |
| Poor (≥30) | 19.7 (2.2) | 1.9 (0.6) | **<0.001** | 8.4 (1.5) | 14.8 (2.9) | 13.1(2.9) | 0.132 |
| *BP (mmHg*) |  |  |  |  |  |  |  |
| Ideal (<120/80 without medication) | 41.1 (2.9) | 37.1 (5.3) |  | 45.9 (4.8) | 36.5 (3.8) | 14.1 (3.6) |  |
| Intermediate (120-139/80-89 or treated to <120/80) | 32.1 (3.0) | 36.5 (5.8) |  | 35.8 (5.1) | 34.9 (3.4) | 26.5 (4.2) |  |
| Poor (≥140/90 or treated to ≥120/80) | 26.7 (2.7) | 26.4 (4.7) | 0.745 | 18.2 (4.0) | 28.7 (3.6) | 59.3 (4.9) | **<0.001** |
| *Total cholesterol (mg/dL)* |  |  |  |  |  |  |  |
| Ideal (<200 without medication) | 80.2 (2.3) | 85.0 (5.9) |  | 89.1 (4.9) | 74.4 (3.8) | 70.1 (4.7) |  |
| Intermediate (200-239 or treated to <200) | 12.9 (1.7) | 12.3 (6.0) |  | 9.7 (5.0) | 16.8 (3.2) | 16.7 (3.4) |  |
| Poor (≥240 or treated to ≥200) | 6.9 (1.7) | 2.7 (0.9) | **0.039** | 1.3 (5.4) | 8.7 (2.6) | 12.9 (3.9) | **0.021** |
| *HbA1c (%)* |  |  |  |  |  |  |  |
| Ideal (<5.7) | 87.4 (1.9) | 95.2 (3.0) |  | 96.8 (2.5) | 88.0 (2.7) | 72.3 (4.3) |  |
| Intermediate (5.7-6.5) | 10.4 (1.7) | 2.8 (1.0) |  | 3.0 (1.1) | 9.1 (2.4) | 18.3 (3.3) |  |
| Poor (≥6.5) | 2.3 (0.8) | 1.9 (0.8) | **0.001** | 1.1 (0.1) | 2.9 (1.0) | 9.4 (2.9) | **<0.001** |
| *Smoking* |  |  |  |  |  |  |  |
| Ideal (never) | 80.4 (2.1) | 68.0 (4.3) |  | 89.7 (2.3) | 58.5 (3.8) | 37.3 (5.1) |  |
| Intermediate (former) | 15.1 (1.7) | 14.5 (2.3) |  | 3.1 (0.8) | 21.9 (2.8) | 52.9 (5.1) |  |
| Poor (current) | 4.6 (1.3) | 17.5 (3.3) | **<0.001** | 7.2 (2.1) | 19.6 (3.5) | 9.7 (2.9) | **<0.001** |
| *Diet (fruit and vegetable servings/week)* |  |  |  |  |  |  |  |
| Ideal (≥20) | 26.7 (3.0) | 16.8 (3.1) |  | 20.2 (3.1) | 25.1 (3.3) | 21.4 (3.5) |  |
| Poor (<20) | 83.2 (3.0) | 73.3 (3.1) | **0.037** | 79.8 (3.1) | 74.9 (3.4) | 78.6 (3.5) | 0.459 |
| *Physical activity* |  |  |  |  |  |  |  |
| Ideal | 76.2 (3.2) | 73.3 (4.6) |  | 78.3 (4.2) | 80.9 (3.0) | 50.2 (5.0) |  |
| Intermediate | 11.6 (2.4) | 17.4 (4.3) |  | 14.8 (3.8) | 10.9 (2.7) | 16.4 (3.4) |  |
| Poor | 12.3 (2.7) | 9.4 (1.8) | 0.341 | 7.0 (2.3) | 8.3 (1.7) | 33.3 (5.0) | **<0.001** |
| C-reactive protein (mg/dL) |  |  |  |  |  |  |  |
| Ideal (≤1) | 30.8 (3.1) | 49.7 (5.6) |  | 48.3 (5.0) | 31.2 (3.4) | 29.2 (4.7) |  |
| Intermediate (1-3 ) | 44.4 (3.0) | 36.8 (4.8) |  | 37.2 (3.4) | 45.5 (3.9) | 38.9 (4.9) |  |
| Poor (>3) | 24.8 (2.4) | 13.5 (4.0) | **0.009** | 14.5 (3.40 | 23.2 (3.3) | 31.9 (4.6) | **0.002** |

*^a^* Boldface indicates statistical significance (p<0.05)

Additional Table 2: Prevalence of ideal cardiovascular health metrics according to age and sex

|  | Weighted population estimates, *proportions (%) (SE)* | | | | | | | |
| --- | --- | --- | --- | --- | --- | --- | --- | --- |
|  |  | Sex | | | Age group (years) | | | |
|  | Total | Female | Male | P value*^a^* | 16-39 | 40-60 | ≥60 | P value*^a^* |
| *No. of ideal AHA CVH metrics+ CRP* |  |  |  |  |  |  |  |  |
| 0 | 0.2 (0.1) | 0.3 (0.2) | 0 |  | 0 | 0.3 (0.3) | 0.7 (0.7) |  |
| 1 | 1.9 (0.7) | 3.0 (1.0) | 0.8 (0.4) |  | 0 | 3.4 (2.2) | 7.5 (2.2) |  |
| 2 | 3.4 (0.7) | 4.7 (1.2) | 2.0 (0.7) |  | 1.5 (0.7) | 2.3 (0.8) | 13.8 (3.8) |  |
| 3 | 10.7 (1.5) | 15.0 (2.4) | 6.3 (1.3) |  | 5.5 (1.8) | 15.4 (2.6) | 25.1 (4.1) |  |
| 4 | 20.4 (2.5) | 20.5 (2.1) | 20.2 (4.3) |  | 15.5 (3.5) | 27.6 (3.5) | 27.5 (4.7) |  |
| 5 | 30.2 (3.4) | 28.1 (3.0) | 32.4 (6.0) |  | 35.2 (5.2) | 25.0 (3.4) | 18.5 (4.1) |  |
| 6 | 20.4 (2.2) | 15.2 (1.8) | 25.8 (4.2) |  | 24.8 (3.6) | 18.4 (2.9) | 4.3 (1.8) |  |
| *7* | 9.7 (2.20 | 8.9 (1.6) | 10.6 (4.0) |  | 12.4 (3.4) | 7.0 (2.2) | 2.5 (1.8) |  |
| 8 | 3.2 (1.0) | 4.4 (1.6) | 1.8 (1.1) | **0.018** | 5.0 (1.6) | 0.6 (0.4) | 0 | <0.001 |
| Mean no. of metrics at ideal CVH level out of 8 | 4.9 (0.08) | 4.7 (0.1) | 5.1 (0.1) | **0.010** | 5.4 (0.1) | 4.5 (0.1) | 3.6 (0.1) | **<0.001** |
|  |  |  |  |  |  |  |  |  |
| Optimal CVH health  (6-8 metrics) | 34.2 (2.9) | 29.0 (2.6) | 39.7 (5.3) |  | 43.8 (4.7) | 26.4 (3.2) | 7.1 (2.4) |  |
| Intermediate health  (3-5 metrics) | 60.3 (3.0) | 63.1 (2.9) | 57.4 (5.3) |  | 54.6 (4.7) | 67.5 (3.6) | 71.6 (4.5) |  |
| Poor health  (0-2 metrics) | 5.4 (0.1) | 7.8 (1.7) | 2.9 (0.9) | **0.025** | 1.6 (0.7) | 6.1 (2.3) | 21.3 (4.2) | **<0.001** |
|  |  |  |  |  |  |  |  |  |
| *No. of ideal AHA CVH metrics* |  |  |  |  |  |  |  |  |
| 0 | 0.3 (0.1) | 0.3 (0.2) | 0.2 (0.2) |  | 0 | 0.3 (0.3) | 1.5 (1.0) |  |
| 1 | 2.0 (0.7) | 3.4 (1.2) | 0.6 (0.4) |  | 0 | 3.4 (2.2) | 8.4 (2.3) |  |
| 2 | 3.9 (0.8) | 4.5 (1.2) | 3.3 (1.1) |  | 1.5 (0.7) | 3.6 (1.2) | 15.4 (4.1) |  |
| 3 | 13.1 (1.6) | 17.5 (2.5) | 8.6 (1.6) |  | 7.3 (2.0) | 19.1 (2.8) | 27.6 (4.2) |  |
| 4 | 30.7 (3.5) | 27.4 (2.9) | 34.1 (6.2) |  | 29.5 (5.5) | 32.0 (3.6) | 33.4 (5.0) |  |
| 5 | 28.5 (2.5) | 25.3 (2.4) | 31.8 (4.6) |  | 33.8 (4.1) | 25.4 (3.3) | 10.6 (2.6) |  |
| 6 | 17.0 (2.5) | 14.8 (1.9) | 19.2 (4.6) |  | 21.5 (3.9) | 13.6 (2.6) | 3.1 (1.9) |  |
| 7 | 4.5 (1.0) | 6.8 (1.7) | 2.2 (1.0) | **0.011** | 6.3 (1.7) | 2.5 (1.1) | 0 | <0.001 |
| Mean no. of metrics at ideal CVH level out of 7 | 4.5 (0.1) | 4.4 (0.1) | 4.6 (0.1) | **0.111** | 4.9 (0.1) | 4.2 (0.1) | 3.3 (0.1) | **<0.001** |
|  |  |  |  |  |  |  |  |  |
| Optimum CVH health  (6-7 metrics) | 22.3 (2.6) | 22.1 (2.4) | 22.4 (4.7) |  | 29.1 (4.1) | 16.4 (3.0) | 3.3 (1.9) |  |
| Intermediate health  (3-5 metrics) | 71.5 (2.7) | 69. 7 (2.7) | 73.3 (4.8) |  | 69.3 (4.2) | 76.0 (3.4) | 72.1 (4.6) |  |
| Poor health  (0-2 metrics) | 6.2 (1.1) | 8.1 (1.7) | 4.3 (1.2) | 0.275 | 1.6 (0.7) | 7.4 (2.5) | 24.7 (4.4) | **<0.001** |

*^a^* Boldface indicates statistical significance (p<0.05)

Additional Table 3: Comparison of population estimates based on weightings from inverse probability of health fair attendance models versus true population statistics

| Characteristic | Weighted Estimate (95%CI) | True Population Estimate |
| --- | --- | --- |
| Lifetime Consumption of Alcohol (%) |  |  |
| Never | 42.3 (36.3-48.5) | 40.9 |
| >5 years ago | 17.2 (13.9-21.0) | 17.5 |
| 1-5 years ago | 11.3 (7.7-16.4) | 9.8 |
| <1 year ago | 29.2 (24.9-34.0) | 31.7 |
| Waist Circumference (cm) | 85.6 (84.1-87.1) | 85.3 |
| Self-Reported HIV Status (%) | 8.3 (6.1-11.1) | 8.7 |
| Self-Reported Happiness (%) |  |  |
| Not happy | 17.0 (13.9-20.7) | 17.3 |
| Fairly happy | 70.6 (64.3-76.2) | 72.4 |
| Very happy | 12.4 (7.5-19.8) | 10.0 |

*^a^* Boldface indicates statistical significance (*p<0.05)

Additional Table 4: Characteristics of Health Fair Attendees versus Non-attendees

| Characteristic | Attendees (n = 829) | Non-attendees (n = 957) | P value*^a^* |
| --- | --- | --- | --- |
| Sex |  |  |  |
| Female | 62.4% | 48.6% | **<0.001** |
| Age (years) | 43.6 (42.3 - 44.8) | 34.1 (33.1 – 35.1) | **<0.001** |
| ≤30 years (%) | 4.3% | 16.5% |  |
| 30-50 years (%) | 65.6% | 69.5% |  |
| >50 years | 30.1% | 14.0% | **<0.000** |
| Formal educational attainment |  |  |  |
| None | 18.5% | 11.7% |  |
| Some primary education | 34.2% | 23.1% |  |
| Completed primary education | 23.5% | 20.9% |  |
| At least secondary education | 23.8% | 44.4% | **<0.001** |
| Self-Reported Health |  |  |  |
| Very bad | 1.4% | 0.7% |  |
| Bad | 26.5% | 13.1% |  |
| Good | 59.6% | 71.1% |  |
| Very Good | 12.6% | 15.0% | **<0.001** |

*^a^* Boldface indicates statistical significance (*p<0.05)

Additional Table 5: Comparison of characteristics of participants with and without complete AHA metrics data

| Characteristic | With complete AHA metrics data (n=785) | Missing AHA metrics data (n=62) | P value*^a^* |
| --- | --- | --- | --- |
| Age (years) | 39.6 (1.2) | 41.0 (1.0) | 0.822 |
| Body Mass Index (kg/m^2^) | 24.3 (0.2) | 24.1 (0.2) | 0.524 |
| Diabetes Mellitus | 1.7 (0.01) | 1.5 (0.02) | 0.428 |
| Hypertension | 13.4 (1.9) | 13.8 (1.2) | 0.833 |
| History of heart failure | 5.1 (1.1) | 4.9 (2.0) | 0.758 |
| History of stroke | 4.6 (1.8) | 4.1 (2.0) | 0.796 |

*^a^* Boldface indicates statistical significance (*p<0.05)

Additional Methods: Description of variables used in the inverse probability of treatment weights (IPTW).

**Food and water insecurity**

*Water insecurity*: this is a composite of several variables related to water:

1. In the past 30 days, how often did you worry about whether your household would have enough water for all of its needs?
2. In the past 30 days, how often did you or any household members collect water for drinking from an undesirable or dirty water source because you could not collect water from a preferred or clean source?
3. In the past 30 days, how often did you or any household members drink water that you thought might not be safe for health?
4. In the past 30 days, how often did you or any household members drink less water than you needed because there was not enough water or because it was too difficult to collect more water?
5. In the past 30 days, how often did you or any household members use less water than you needed because there was not enough water or because it was too difficult to collect more water?
6. In the past 30 days, how often was there no water at all in your household because it was too difficult to collect more water?
7. In the past 30 days, how often did you or any household members go to sleep at night thirsty because there was not enough water?
8. In the past 30 days, how often did you feel angry or frustrated about not having enough water for the household?

Each question was answered on a 0-3 scale, 0 meaning never, 1 meaning rarely, 2 meaning sometimes, and 3 meaning often. Total score was calculated by summing all questions. This total score was then quintiled.

*Food insecurity*: this is a composite of several variables related to food:

1. In the past 30 days, how often was there no food at all in your household because you lacked money to purchase more?
2. In the past 30 days, how often did you or any household members go to sleep at night hungry because there was not enough food?
3. In the past 30 days, how often did you or any household members go a whole day without eating anything because there was not enough food?

Each question was answered on a 0-3 scale, 0 meaning never, 1 meaning rarely, 2 meaning sometimes, and 3 meaning often. Total score was calculated by summing all questions. This total score was then quintiled.

**Alcohol use**

This variable measured whether or not the survey participant was a heavy drinker. The heavy drinker variable was created using three measures of alcohol use, including bingeing (“In the past year, did you ever take 6 or more drinks in a single morning, afternoon, or night?”), spending on alcohol (“In the past 30 days, did you yourself spend more than 25,000 USh on any kind of alcohol?”), and time spent intoxicated (“In the past 30 days, did you experience drunkenness or intoxication on 3 or more of those days?”). A “Yes” answer to any of those three questions classified the respondent as a heavy drinker.

**Household asset ownership**

The household asset index was created through principal components analysis, including ownership of land (number of plots), a radio, a lantern, a bike, a television, an electric iron, a *boda-boda* (motorcycle), a refrigerator, a stove, a car, a ventilated improved pit latrine, cement walls, and cement floors. The household asset index did not include variables with many missing observations (number of cows, number of goats, number of chickens, ownership of a mobile phone, number of rooms in house, ownership of a rainwater harvesting tank). The household asset index was then quintiled.

**Sex**

Self-reported sex of the survey respondent.

**Age**

Age of the survey respondent categorized as 17, 18-25, 26-35, 36-45, 46-55, or 56 years and older.

**Marital status**

Self-reported marital status of the survey respondent (married/cohabitating, single/never married or separated/divorced/widowed).

**Village of residence**

Village of residence of the survey respondent (Buhingo, Bushenyi, Nyamikanja I, Bukuna II, Nyakabare, Bukuna I, Rwembogo, or Nyamikanja II).

**Distance from the HF**

This variable measured the distance between the survey respondent’s village and the health fair site. There were three different health fair sites, each occurring on a different day. Using the registration day of the respondent to determine which health fair site they attended, and the coordinates of the respondent’s village, distance between respondent village and health fair site was calculated using Stata’s geodist command. The latitude and longitude of all three health fair sites were averaged to compute average village to health fair distances, and this was used to fill in the missing values for respondents who did not attend the health fair.

**Difference between the altitude of the household residence and the altitude of the HF**

This variable measured the altitude between the survey respondent’s village and the health fair site. There were three different health fair sites, each occurring on a different day. Using the registration day of the respondent to determine which health fair site they attended, and the altitude of the respondent’s village, altitude between respondent village and health fair site was calculated. The altitudes of all three health fair sites were averaged to compute average village to health fair altitude differences, and this was used to fill in the missing values for respondents who did not attend the health fair.

**Educational attainment**

Educational attainment category of the survey respondent (none; some primary, P1-P6; completed primary, P7-P8; more than primary, S1-S6, vocation, or university).

**Self-reported HIV status**

Self-reported HIV status of the survey respondent (positive or negative).

**Self-reported overall health**

Self-reported overall health of the survey respondent (very good, good, bad, or very bad).

**Social network size**

This variable measured the survey respondent’s social network size. The survey respondent was asked to name up to six people (18 years or older) in five categories (up to 30 people total) that they share some sort of social relationship with. The categories included people with whom the survey respondent spent time for leisure, enjoyment, or relaxation; people with whom the survey respondent discussed any kind of money matters; people to whom the survey respondent had gone to for emotional support; people with whom the survey respondent discussed any kind of health issue; and people with whom the survey respondent shared, borrowed, received, or exchanged any food. The number of people named by the survey respondent was used as an approximate measure for social network size.

**Index of social participation**

This variable measured the survey respondent’s social participation through counting the number of social groups the respondent is a part of. This included vocational groups, positive living groups (for HIV positive people), local council committees, water committees, VHT groups, NAADS groups, revolving fund/SACCO/any other registered savings groups, church or other religious groups, women’s groups, and gardening committees.
